# Changed health behavior improves subjective well-being and vice versa in a follow-up of 9 years

**DOI:** 10.1186/s12955-022-01972-4

**Published:** 2022-04-21

**Authors:** Säde Stenlund, Heli Koivumaa-Honkanen, Lauri Sillanmäki, Hanna Lagström, Päivi Rautava, Sakari Suominen

**Affiliations:** 1 Department of Public Health, University of Turku, 20014 Turku, Finland; 2 Research Services, Turku University Hospital, 20014 Turku, Finland; 3 Institute of Clinical Medicine, Psychiatry, University of Eastern Finland, 70211 Kuopio, Finland; 4 Mental Health and Wellbeing Center, Kuopio University Hospital, 70029 Kuopio, Finland; 5 Department of Public Health, University of Helsinki, 00014 Helsinki, Finland; 6 Centre for Population Health Research, University of Turku and Turku University Hospital, 20014 Turku, Finland; 7 School of Health Sciences, University of Skövde, 54128 Skövde, Sweden

**Keywords:** Health behavior, Subjective well-being, Health behavior change, Life satisfaction, Follow-up, Longitudinal

## Abstract

**Background:**

Previous research on health behavior and subjective well-being has mainly focused on interindividual differences or explored certain domains of health behavior. Good health behavior and subjective well-being at baseline can predict each other after a follow-up. In the present cohort study, we explored the outcomes of change for an individual i.e., how changed health behavior is reflected in subsequent subjective well-being and vice versa.

**Methods:**

Data (n = 10,855) originates from a population-based Health and Social Support (HeSSup) study on working-age Finns in 2003 and 2012. A composite measure of health behavior included physical activity, dietary habits, alcohol consumption, and smoking status (range 0–4, worst–best) and a composite measure of subjective well-being (with reversed scoring) included three life assessments, i.e., interest, happiness, and ease in life, and perceived loneliness (range 4–20, best–worst). Different multiple linear regression models were used to study how changes in health behavior predict subjective well-being and the opposite, how changes in subjective well-being predict health behavior.

**Results:**

A positive change in health behavior from 2003 to 2012 predicted better subjective well-being (i.e., on average 0.31 points lower subjective well-being sum score), whereas a negative change predicted poorer subjective well-being (i.e., 0.37 points higher subjective well-being sum score) (both: *p* < 0.001) compared to those study subjects who had no change in health behavior. Similarly, when a positive and negative change in subjective well-being was studied, these figures were 0.071 points better and 0.072 points worse (both: *p* < 0.001) health behavior sum score, respectively. When the magnitude of the effect of change was compared to the range of scale of the outcome the effect of health behavior change appeared stronger than that of subjective well-being.

**Conclusion:**

Changes in health behavior and subjective well-being have long-term effects on the level of the other, the effect of the first being slightly stronger than vice versa. These mutual long-term benefits can be used as a motivator in health promotion on individual and societal levels.

**Supplementary Information:**

The online version contains supplementary material available at 10.1186/s12955-022-01972-4.

## Introduction

In general, subjective well-being (SWB) comprise of both a cognitive component, i.e. life satisfaction, and an affective component (i.e. both positive and negative affect) [1]. Thus, SWB measures can vary by their components or items. The cross-sectional association between domains of health behavior and different measures of SWB is well-established [2–5]. However, the bidirectional nature of this association has been addressed to require further research [6].

Changes in health behavior with respect to subsequent SWB and vice versa have been studied, but only in unidirectional settings. Increases in physical activity have co-occurred with changes in life satisfaction on a day-to-day basis [7] and in three major Socio-Economic Panels in Germany, Britain and Australia [8]. In the general adult population, an increase in the consumption of fruit and vegetable resulted in higher life satisfaction in a 5-year follow-up in UK [9] and in a 2-year follow-up in Australia [10].

On the other hand, changes in SWB have also been studied with respect to health behavior. In a 5-year follow-up of cardiovascular patients, increases in positive affect co-occurred with physical activity improvements, but not with changes in smoking status. However, baseline positive affect did not predict better health behavior, when baseline health behavior was controlled [11]. In a 4-week web-based follow-up, positive changes in life satisfaction and mood were both associated with decreases in dependence to smoking and higher rates of smoking cessation, whereas positive changes in life satisfaction with reduction in smoking, and positive changes in mood with decrease in feeling prisoner to cigarettes as well as smaller odds in smoking relapse [12].

Even if previous research is sparse on the role of change in the unidirectional setting between heath behavior and measures of SWB, there is a complete lack of studies on the role of their changes measured in bidirectional settings with the same data and with the same measures. Thus, the aim of the present study was to explore how change in a composite measure of health behavior predicts a subsequent composite measure of SWB and vice versa in a population-based sample of 10,000 working-age Finns.

## Methods

**Fig. 1:**
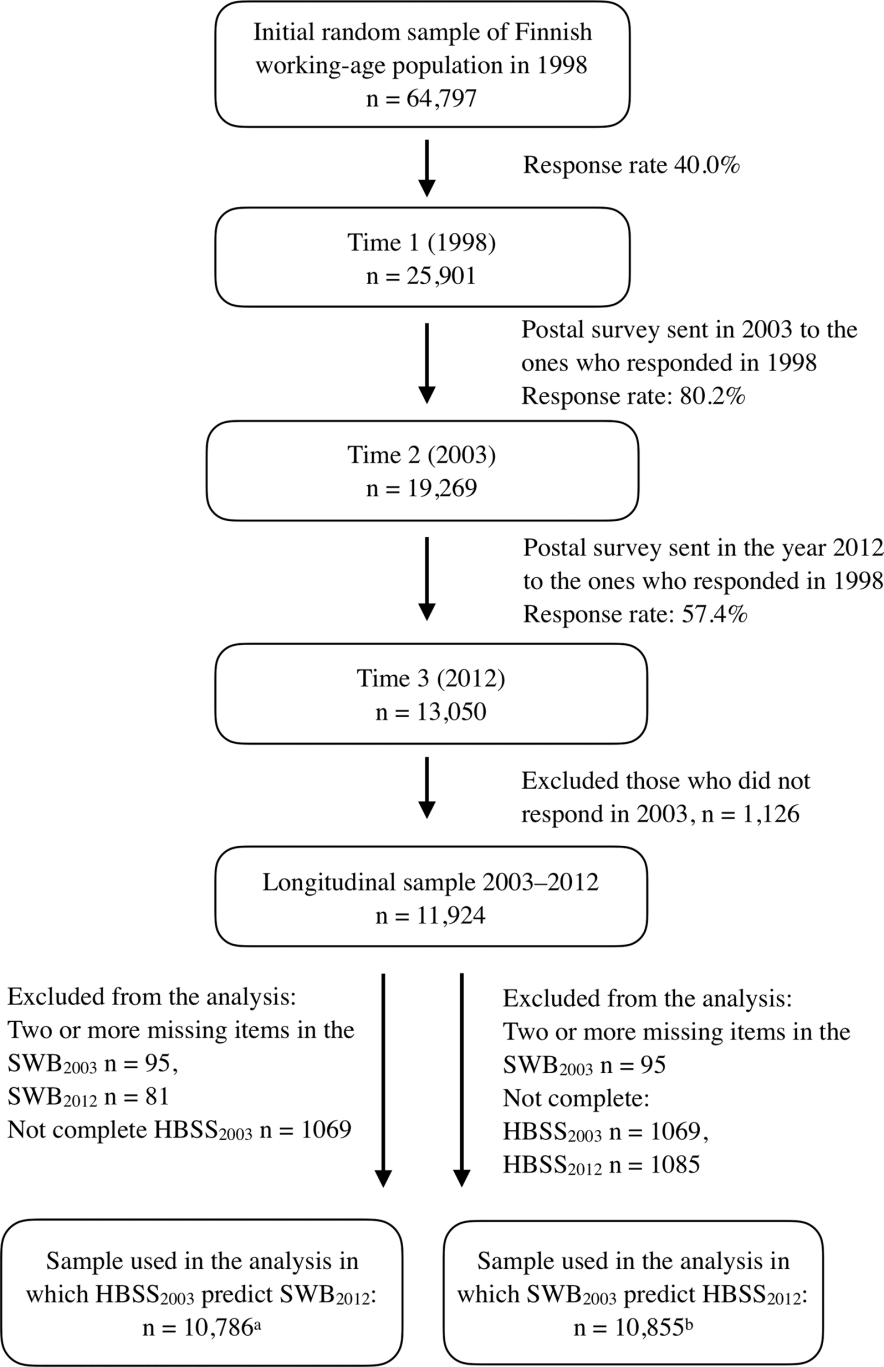
Inclusion and exclusion criteria for the study population. From Health and Social Support (HeSSup) prospective population-based follow-up study. ^a^n = 9986–10,786, due to missing responses on covariates. ^b^n = 9986–10,855, due to missing responses on covariates. HBSS_2003_ = Health behavior sum score i.e., no. of protective health behaviors in 2003 (baseline). HBSS_2012_ = Health behavior sum score i.e., no. of protective health behaviors in 2012 (follow-up). SWB_2003_ = Subjective well-being in 2003 (baseline). SWB_2012_ = Subjective well-being in 2012 (follow-up)

Data (n = 10,855) originated from the second (in 2003) and third (in 2012) waves of a population-based Health and Social Support (HeSSup) study on working-age Finns. Unlike the first wave (in 1998), the questionnaires in these two waves included identical items in an identical or almost identical order for both SWB and health behavior. For details on response rates and the inclusion of participants see Fig. 1. Details of attrition of participants can be found elsewhere [13].

### Measures

A continuous health behavior sum score (HBSS, range 0–4) is a count of the number of beneficial health behaviors including physical activity, dietary habits, alcohol consumption, and smoking status. P*hysical activity* performed in leisure time or in commuting was first converted into a Metabolic Equivalent Task (MET). A MET score ≥ 2 corresponding to 30 min walking per day was the cut-off value for beneficial behavior and provided one point in the HBSS. *Dietary habits* were assessed by a non-validated index (range 0–100), but formed in compliance with the Nordic nutritional recommendations [14]. Each choice, if being in line with the recommendations, provided one point in the dietary index according to the following cut-off points: dark bread (≥ 2/day); fat free milk (≥ 1/day); pastries/ sweets, sausages, red meat or chicken/ turkey (each ≤ 1–2/week); fish (≥ 1–2/week); fresh fruits and berries (≥ 2/day); vegetables (≥ 2/day); alcohol use (< 10 g women, 20 g men/day). Then, the sum score ranging from 0 to 10 was multiplied by 10 to give a percentage of compliance to recommendations [15]. Lastly, a cut-off value above median in this dietary index (≥ 60) provided one point for HBSS. *Alcohol consumption* was dichotomized according to Finnish guidelines [16] where risky consumption for women is ≥ 140 g/week and for men ≥ 280 g/week. Then, the values lower than the relevant cut-off provided one point for HBSS. *Smoking* status was dichotomized into current smokers vs. non-smokers in combination with former-smokers. The latter choice provided one point in HBSS.

Categorical *change in health behavior* (i.e., positive, neutral, and negative change) was measured by the change in the health behavior sum score (HBSSchange) during follow-up (i.e. the difference between HBSS_2012_ and HBSS_2003_). For evaluating change, individuals reporting zero or one beneficial health behaviors were combined into one group due to the small number of participants. Participants were categorized into three groups according to the direction of change in the HBSS during follow-up: positive, neutral, and negative change.

SWB was measured with the continuous four-item life satisfaction scale (range 4–20) [17, 18] where a lower score indicates better subjective well-being. It has three life assessments (i.e., interest, happiness, and ease in life) representing the cognitive component of SWB [1]. The fourth item is perceived loneliness, which is not typical for a life satisfaction scale, but reflects social well-being. The wordings of the life assessments were “*Do you feel that your life at the moment is* …”, and for the perceived loneliness “*Do you feel that at the moment you are …?* The responses were scored as follows: very interesting/ happy/ easy/ not at all lonely = 1; fairly interesting/ happy/ easy = 2; cannot say = 3; fairly boring/ unhappy/ hard/ lonely = 4; very boring/ unhappy/ hard/ lonely = 5 [18, 19]. Previously, the life satisfaction scale has often had three categories: satisfied (score = 4–6); intermediate group (score = 7–11, within ± 1 SD from the mean); dissatisfied (score = 12–20). In the present study, the intermediate group was divided at the mean to create four groups of SWB: high (score = 4–6), high intermediate (score = 7–8), low intermediate (score = 9–11), and low (score = 12–20).

The *change in subjective well-being* (SWBchange) during follow-up was assessed based on the direction of change between SWB groups with three categories (as in HBSSchange): positive, neutral, or negative change (each: yes/no). Thus, the SWBchange indicated the direction of change from one SWB group to another during the follow-up according to the difference in the group levels of SWB_2012_ and SWB_2003_, not in their continuous scores.

Factors potentially affecting both SWB and health behavior were included as covariates according to groupings based on the initial random sampling (age) and previous publications of the data. Participants represented four *age* groups in 2003: 25–29 years (group 1), 35–39 years (group 2), 45–49 years (group 3), and 55–59 years (group 4). *Education* was also categorized into four groups: (1) no professional education; (2) vocational course/school/apprenticeship contract; (3) college; (4) university degree/university of applied sciences. *Health status* was categorized according to count of the self-reported diseases into three groups: 0, 1, ≥ 2 diseases. Disease count is commonly used due to its simplicity and the ease of data ascertainment. Even if it does not consider the severity of disease, it is linked with mortality and various health adversities [20]. The complete list of 35 diseases can be found in the Additional file 1. *Negative life events* before the follow-up survey could affect both SWB and health behavior. From a list of 21 life events in 2007–2012, participants reported burdensome and extremely burdensome life events [21] which were then transformed into a trichotomized covariate: 0, 1, ≥ 2 major negative life events. Details of the life events and the item in the survey can be found in the Additional file 1.

### Statistical analyses

Linear regression models were used to analyze:

1. The effect of the categorical HBSSchange on the association between baseline HBSS (continuous HBSS_2003_) and follow-up SWB (continuous SWBscore_2012_)
2. The effect of categorical SWBchange on the association between baseline SWB (continuous SWBscore_2003_) and follow-up HBSS (continuous HBSS_2012_).

As already described, both HBSSchange and SWBchange were measured by the difference between their baseline and end of follow-up grouping divided into three categories (i.e. positive, neutral, and negative change). The first digit of the adjustment models identified the direction (1: from HBSS_2003_ to SWB_2012;_ 2: from SWB_2003_ to HBSS_2012_) and the second digit was for the adjustment factors. Thus, the Model 1.1. from HBSS_2003_ to SWB_2012_ was adjusted for age, gender, education, and disease count (Table 3). The additional adjustments were for either HBSSchange (Model 1.2), or SWB_2003_ (Model 1.3) or both (Final model 1.4.). The Model 1.4 was then further adjusted with negative life events to create Model 1.5 (For clarification see Table 3). To study the effect of change without baseline level, HBSSchange was made the predictor instead of baseline HBSS_2003_ to create Model 1.6, which is otherwise parallel to Model 1.1. The Model 1.7 was created by adding also SWB_2003_ in Model 1.6. (Table 3).

In the opposite direction (from SWB_2003_ to HBSS_2012_) (Table 5), the Model 2.1 was adjusted for age, gender, education, and disease count as in Model 1.1., but it had also the SWB_2003_*education interaction term (Table 5). The additional adjustments were for either SWBchange (Model 2.2) or HBSS_2003_ (Model 2.3) or both (Final model 2.4). The Model 2.4 was then further adjusted with negative life events to create Model 2.5. To study the effect of change without baseline level, SWBchange was made the predictor instead of baseline SWB_2003_ and the interaction term SWB_2003_*education was converted to SWBchange*education to create Model 2.6 from the Model 2.1. The model 2.7 was created by adding also HBSS_2003_ in the Model 2.6. (Table 5). Data were analyzed with SAS software (version 9.4; SAS Institute Inc. Cary, NC, USA 2016).

To be able to compare the effects of the SWBchange and HBSSchange on their outcomes i.e. HBSS_2012_ (range 0–4 with 4 steps) and SWB_2012_ (range 4–20 with 16 steps), the HBSS_2012_ should be rescaled. Thus, the estimate for SWBchange is multiplied by 4 (4 × 4 = 16) to be comparable for the estimate of HBSSchange.

## Results

During the 9-year follow-up the average HBSS for the entire study population improved from 2.87 to 2.99. A positive HBSSchange was observed in 30.5% (n = 3100), no change in 48.3% (n = 4913), and deterioration in 21.2% (n = 2160). For details, see Table 1. Average SWB improved from score 8.53 to 8.37 (lower scores indicating better SWB). Its greatest improvements were observed in the oldest age group and in individuals experiencing no major negative life events. A positive SWBchange was observed in 30.2% of the study population (n = 3546), neutral change in 44.0% (n = 5172), and negative change in 25.8% (n = 3034). For details, see Table 2. Details of the distribution of individual health behaviors at baseline can be found elsewhere [13].

**Table 1:** Health behavior sum score at baseline (HBSS_2003_) and after follow-up (HBSS_2012_) as well as its changes (HBSSchange) by subgroup, as percentages (numbers), unless otherwise stated. Results of the Finnish population-based Health and Social Support Study

Variable	Category	Share of the study population	HBSS_2003_ mean (SD)	HBSS_2012_mean (SD)	Positive HBSSchange	Neutral HBSSchange	Negative HBSSchange
		100 (10,855)	2.87 (0.90)	2.99 (0.99)	30.5 (3100)	48.3 (4913)	21.2 (2160)
Age (2003)	25–29	20.6 (2234)	2.91 (0.84)	3.04 (0.85)	31.9 (674)	46.6 (985)	21.6 (457)
	35–39	20.7 (2246)	2.82 (0.89)	2.96 (0.91)	31.6 (673)	48.0 (1024)	20.4 (436)
	45–49	26.6 (2885)	2.82 (0.94)	2.94 (0.94)	29.9 (804)	48.9 (1317)	21.2 (572)
	55–59	32.2 (3490)	2.91 (0.91)	3.01 (0.88)	29.4 (949)	49.1 (1587)	21.5 (695)
Gender	Male	36.2 (3925)	2.66 (0.92)	2.82 (0.92)	32.5 (1177)	47.1 (1709)	20.4 (739)
	Female	63.8 (6930)	2.98 (0.87)	3.09 (0.87)	29.4 (1923)	48.9 (3204)	21.7 (1421)
Education (2003)	No professional education	12.0 (1302)	2.67 (0.93)	2.81 (0.92)	31.1 (376)	48.7 (590)	20.2 (245)
	Vocational school	29.0 (3136)	2.71 (0.92)	2.83 (0.94)	31.5 (922)	47.0 (1379)	21.5 (631)
	College	39.0 (4218)	2.91 (0.89)	3.03 (0.89)	30.2 (1195)	48.6 (1924)	21.3 (844)
	University	19.9 (2154)	3.13 (0.81)	3.23 (0.77)	29.2 (591)	49.3 (1000)	21.5 (436)
Diseases (2003)	0	17.9 (1931)	2.94 (0.86)	3.06 (0.86)	30.8 (557)	48.0 (869)	21.2 (384)
	1	23.3 (2522)	2.89 (0.88)	3.04 (0.89)	31.2 (740)	49.2 (1167)	19.5 (464)
	2 or more	58.8 (6355)	2.84 (0.92)	2.95 (0.91)	30.0 (1787)	48.0 (2856)	22.0 (1306)
Subjective well-being (2003)	High	25.04 (2962)	3.03 (0.85)	3.12 (0.84)	29.0 (730)	49.2 (1238)	21.9 (551)
	High intermediate	34.18 (4043)	2.89 (0.88)	3.01 (0.87)	30.4 (1052)	48.6 (1684)	21.1 (730)
	Low intermediate	22.61 (2674)	2.85 (0.89)	2.98 (0.88)	31.4 (718)	47.6 (1086)	21.0 (480)
	Low	18.18 (2150)	2.63 (0.97)	2.77 (0.98)	31.2 (574)	47.6 (876)	21.2 (391)
Negative life events	0	39.4 (5135)	2.88 (0.90)	2.98 (0.89)	34.4 (1286)	51.3 (1915)	14.3 (533)
	1	25.3 (3299)	2.90 (0.89)	3.02 (0.89)	35.0 (800)	49.6 (1133)	15.4 (353)
	2 or more	35.4 (4616)	2.83 (0.92)	2.95 (0.93)	34.9 (1010)	46.3 (1341)	18.8 (545)

HBSS, Health behavior sum score; HBSSchange, Change in health behavior sum score during follow-up; Diseases (2003), Count of the self-reported diseases in 2003 (baseline). List of diseases is in the Additional file 1

**Table 2:** Subjective well-being at baseline (SWBscore_2003_) and after follow-up (SWBscore_2012_) as well as its changes (SWBchange) by subgroup, as percentages (numbers), unless otherwise stated. Results of the Finnish population-based Health and Social Support Study

Variable	Category	Share of the study population	SWBscore_2003_ mean (SD)	SWBscore_2012_ mean (SD)	Positive SWBchange	Neutral SWBchange	Negative SWBchange
		100 (10,855)	8.53 (3.20)	8.37 (3.18)	30.2 (3546)	44.0 (5172)	25.8 (3034)
Age (2003)	25–29	20.6 (2234)	8.47 (3.15)	8.43 (3.15)	31.6 (773)	37.6 (918)	30.8 (752)
	35–39	20.7 (2246)	8.58 (3.28)	8.67 (3.38)	27.9 (675)	43.1 (1045)	29.0 (703)
	45–49	26.6 (2885)	8.64 (3.31)	8.57 (3.31)	28.5 (896)	45.6 (1435)	25.9 (815)
	55–59	32.2 (3490)	8.45 (3.06)	7.96 (2.90)	32.1 (1202)	47.4 (1774)	20.4 (764)
Gender	Male	36.2 (3925)	8.58 (3.18)	8.36 (3.16)	31.2 (1357)	44.3 (1926)	24.5 (1067)
	Female	63.8 (6930)	8.51 (3.20)	8.38 (3.20)	29.6 (2189)	43.9 (3246)	26.6 (1967)
Education (2003)	No professional education	12.0 (1302)	8.89 (3.33)	8.58 (3.26)	29.5 (417)	46.3 (654)	24.2 (342)
	Vocational school	29.0 (3136)	8.76 (3.30)	8.54 (3.27)	30.2 (1013)	45.3 (1521)	24.5 (821)
	College	39.0 (4218)	8.40 (3.15)	8.24 (3.07)	29.9 (1362)	43.1 (1964)	27.1 (1236)
	University	19.9 (2154)	8.24 (3.01)	8.08 (3.01)	31.0 (737)	42.6 (1012)	26.3 (625)
Diseases (2003)	0	17.9 (1931)	7.84 (2.73)	7.91 (2.85)	27.4 (579)	45.2 (957)	27.4 (581)
	1	23.3 (2522)	8.12 (2.94)	7.98 (2.89)	30.6 (834)	42.5 (1160)	26.9 (733)
	2 or more	58.8 (6355)	8.91 (3.37)	8.60 (3.31)	30.6 (2115)	44.3 (3035)	24.9 (1705)
Health behavior sum score (2003)	0 or 1	7.0 (763)	9.90 (3.87)	9.53 (3.73)	30.4 (227)	43.7 (326)	25.9 (193)
	2	24.5 (2654)	9.02 (3.37)	8.78 (3.35)	30.9 (810)	43.8 (1148)	25.4 (665)
	3	42.4 (4602)	8.38 (3.06)	8.22 (3.07)	29.8 (1355)	44.4 (2021)	25.8 (1174)
	4	26.1 (2836)	8.03 (2.91)	7.85 (2.77)	30.6 (856)	42.7 (1195)	26.8 (749)
Negative life events	0	39.4 (5135)	8.16 (2.99)	7.68 (2.67)	31.6 (1470)	46.8 (2174)	21.6 (1004)
	1	25.3 (3299)	8.40 (3.05)	8.15 (3.01)	30.7 (930)	44.8 (1356)	24.5 (742)
	2 or more	35.4 (4616)	9.07 (3.45)	9.29 (3.58)	28.1 (1146)	40.3 (1642)	31.6 (1288)

SWBscore, Subjective well-being score; SWBchange, Change in subjective well-being subgroup during follow-up; Diseases (2003), Count of the self-reported diseases in 2003 (baseline). List of diseases is in the Additional file 1.

### HBSSchange predicting SWB_2012_

HBSS_2003_ predicted the SWB_2012_ in each model (Table 3), the β being – 0.35 (p < 0.001, Akaike information criterion (AIC) = 48,300) in the final model 1.5 (adjusted for age, gender, education, disease count, HBSSchange, SWB_2003_ and negative life events). A positive HBSSchange predicted 0.31 (*p* < 0.001) points lower SWBscore_2012_ (i.e., better SWB) and a negative change predicted 0.37 (*p* < 0.001) points higher SWBscore_2012_ after follow-up compared to those with no change in the HBSS_2003_ during follow-up (Table 4). In addition, every one point increase in the HBSS_2003_ (β = 0.35; *p* < 0.001) resulted in a 0.35 points lower SWBscore_2012_ (indicating better SWB) and every higher point in the SWBscore_2003_ (β = 0.42; *p* < 0.001) resulted in a 0.42 points higher SWBscore_2012_. With the reference group being at least two major negative life events in 2007–2012, having one or none major negative life event resulted separetely analyzed in overall 0.75 (*p* < 0.001) and 1.08 (*p* < 0.001) points lower SWBscore_2012_ (indicating better SWB), respectively. Further, lower disease count in 2003 (*p* < 0.001) resulted in better SWB_2012_ compared to those with at least two diseases. Age as a covariate (*p* < 0.001) resulted in a U-shaped pattern in SWB_2012_ (age group 2 having worst SWB_2012_). In addition, education was a significant covariate (*p* = 0.038) but not gender. Using HBSSchange as a predictor instead of HBSS_2003_ in Model 1.1. (Model 1.6) or adding baseline SWBscore_2003_ in it (Model 1.7), did not improve the AIC or statistical significance of covariates. (Table 3).

**Table 3:** Linear regression models in which baseline health behavior (HBSS_2003_) or its change (HBSSchange) predicts follow-up subjective well-being (SWBscore_2012_)

Model	HBSS_2003_ β *p*-value (standard error)	HBSSchange *p*-value	SWBscore_2003_ β *p*-value (standard error)	Age *p*-value	Gender *p*-value	Education *p*-value	Diseases *p*-value	Negative life events *p*-value	AIC
Model 1.0: Crude model, no covariates	− 0.51	–	–	–	–	–	–	–	55,200
	< .001								
	(0.033)								
Model 1.1: Model 1.0 + Age, gender, education, diseases	− 0.47	–	–	< 0.001	0.11	< 0.001	< 0.001	–	54,500
	< .001								
	(0.034)								
Model 1.2: Model 1.1 + HBSSchange	− 0.64	< 0.001	–	< 0.001	0.038	0.003	< 0.001	–	51,100
	*p* < .001								
	(0.040)								
Model 1.3: Model 1.1 + SWBscore_2003_	− 0.24	–	0.44	< 0.001	0.26	0.023	< 0.001	–	51,800
	< .001		< .001						
	(0.031)		(0.0086)						
Model 1.4: Model 1.1 + HBSSchange + SWBscore_2003_	− 0.36	< 0.001	0.44	< 0.001	0.10	0.10	< 0.001	–	48,600
	< .001		< .001						
	(0.036)		(0.0088)						
**Final model** 1.5: Model 1.4 + Negative life events	− 0.35	< 0.001	0.42	< 0.001	0.45	0.038	< 0.001	< 0.001	48,300
	< .001		< .001						
	(0.036)		(0.0088)						
Model 1.6: HBSSchange + Age, gender, education, diseases	–	.018	–	< .001	0.42	< 0.001	0.002	–	51,300
Model 1.7: HBSSchange + SWBscore_2003_ + Age, gender, education, diseases	–	0.001	0.45	< 0.001	0.88	< 0.001	< .001	–	48,700
			< .001						
			(0.0088)						

Results of Finnish population-based Health and Social Support study

HBSS, Health behavior sum score indicating number of protective health behaviors; HBSSchange, Change in health behavior sum score during follow-up (categorical variable, c.f. Table 4); SWBscore, Subjective well-being score with reversed scoring (lower scores indicating better SWB)

**Table 4:** Estimates of the linear regression model 1.5 in which health behavior (HBSS_2003_) predicts subjective well-being (SWBscore_2012_) Results of Finnish population-based Health and Social Support study

Category	Estimate	Standard error	*p*-value
*Intercept*	6.31	0.17	< 0.001
*HBSS*_*2003*_	–0.35	0.036	< 0.001
*HBSSchange*			
Positive	–0.31	0.068	< 0.001
Neutral	Reference		
Negative	0.37	0.072	< 0.001
*SWBscore*_*2003*_	0.42	0.0088	< 0.001
*Gender*			
Male	0.046	0.059	0.10
Female	Reference		
*Age (2003)*			
25–29	0.35	0.082	< 0.001
35–39	0.47	0.080	< 0.001
45–49	0.33	0.074	< 0.001
55–59	Reference		
*Education (1998)*			
No professional education	0.053	0.094	0.57
Vocational school	Reference		
College	–0.16	0.068	0.019
University or higher	–0.11	0.081	0.17
*Diseases (2003)*			
0	–0.22	0.077	0.005
1	–0.29	0.069	< 0.001
*Negative life events (2007–2012)*			
0	–1.08	0.067	< 0.001
1	–0.75	0.071	< 0.001
2 or more	Reference		

HBSS, Health behavior sum score i.e. no. of protective health behaviors; HBSSchange, Change in health behavior sum score during follow-up; SWBscore, Subjective well-being score (lower scores indicating better SWB)

### SWBchange predicting HBSS

In the final model 2.4 (adjusted for age, gender, education, disease count, HBSSchange and HBSS_2003_) β was –0.025 (*p* < 0.001, AIC = 23,000). A positive SWBchange resulted in 0.071 (*p* < 0.001) points higher HBSS_2012_ and a negative change 0.072 points lower HBSS_2012_ compared to the ones who experienced a neutral SWBchange during follow-up. Every point towards better SWB_2003_ (decrease in score) improved HBSS_2012_ by 0.025 points, and every additional point in HBSS_2003_ improved the HBSS_2012_ by 0.48 points. Better HBSS_2012_ was observed in women (*p* < 0.001) (vs. men) and those with no diseases (*p* < 0.03) (vs. ≥ 2 diseases). Further, having a university level education had a statistically significant effect on the interaction SWB_2003_*education (*p* = 0.005). Age appeared to have a U-shaped effect on subsequent health behavior, but it lost its significance when covariates were added. Education by itself was not a significant covariate. For details of different models, see Table 5, and for final model 2.4, see Table 6. The model did not improve when adjusting for negative life events (*p* = 0.52) or when SWBscore_2003_ was replaced by SWBchange and the interaction SWBchange*education included in the Model 2.6 (Table 5).

**Table 5:** Linear regression models in which baseline subjective well-being (SWBscore_2003_) or its change (SWBchange) predicts follow-up health behavior (HBSS_2012_)

Model	SWBscore_2003_ β *p*-value (standard error)	SWBchange *p*-value	HBSS_2003_ β *p*-value	Age *p*-value	Gender *p*-value	Education *p*-value	Diseases *p*-value	SWB_2003_* Education *p*-value	Negative life events *p*-value	AIC
Model 2.0: Linear model, no covariates	− 0.038	–	–	–	–	–	–	–	–	28,000
	< .001									
	(0.0027)									
Model 2.1: Model 2.0 + Age, gender, education, diseases, SWB_2003_*education	− 0.040	–	–	< 0.001	< 0.001	0.041	0.002	0.003	–	27,300
	< .001									
	(0.0048)									
Model 2.2: Model 2.1 + SWBchange	− 0.049	< .001	–	< 0.001	< 0.001	0.024	0.007	0.009	–	27,200
	< .001									
	(0.0049)									
Model 2.3: Model 2.1 + HBSS_2003_	− 0.019	–	0.49	0.040	< 0.001	0.55	0.020	0.003	–	23,100
	< .001		*p* < .001							
	(0.0031)		(0.0088)							
**Final model** 2.4: Model 2.1 + SWBchange + HBSS_2003_	− 0.025	< .001	0.48	0.093	< 0.001	0.49	0.040	0.005	–	23,000
	< .001		*p* < .001							
	(0.0045)		(0.0088)							
Model 2.5: Model 2.4 + negative life events	− 0.026	< .001	0.48	0.079	< 0.001	0.50	0.036	0.005	0.52	23,000
	< .001		*p* < .001							
	(0.0045)		(0.0088)							
Model 2.6: SWBchange + Age, gender, education, diseases, SWBchange*education	–	.070	–	< 0.001	< 0.001	< 0.001	< 0.001	0.72^b^	–	27,400
Model 2.7: Model 2.6 + HBSS_2003_	–	< .001	0.49	0.022	< 0.001	< 0.001	0.001	< 0.55^b^	–	23,000
			*p* < .001							
			(0.0088)							

HBSS, Health behavior sum score i.e. no. of protective health behaviors; the SWBscore, Subjective well-being score (lower scores indicating better SWB); SWBchange, Change in health behavior sum score during follow-up; SWBchange*education, Interaction term in the model 2.6 used because SWB_2003_ was excluded

**Table 6:** Linear regression model 2.4 in which subjective well-being (SWBscore_2003_) predicts health behavior (HBSS_2012_)

Category	Estimate	Standard error	*p*-value
*Intercept*	1.81	0.053	< 0.001
*SWBscore*_*2003*_	− 0.025	0.0045	< 0.001
*SWBchange*			
Positive	0.071	0.019	< 0.001
Neutral	Reference		
Negative	− 0.072	0.019	< 0.001
*HBSS*_*2003*_	0.48	0.0088	< 0.001
*Gender*			
Male	− 0.10	0.016	< 0.001
Female	Reference		
*Age (2003)*			
25–29	− 0.024	0.023	0.30
35–39	− 0.033	0.022	0.14
45–49	− 0.051	0.020	0.013
55–59	Reference		
*Education (1998)*			
No professional education	0.012	0.075	0.87
Vocational school	Reference		
College	0.061	0.053	0.25
University or higher	− 0.023	0.064	0.72
*Diseases (2003)*			
0	0.046	0.021	0.030
1	0.037	0.019	0.055
2 or more	Reference		
*SWB*_2003_*education			
No professional education	− 0.0019	0.0079	0.81
Vocational school	Reference		
College	0.0017	0.0057	0.76
University or higher	0.022	0.0071	0.0016

HBSS, Health behavior sum score i.e. no. of protective health behaviors; SWBscore, Subjective well-being score (lower scores indicating better SWB); SWBchange, Change in health behavior sum score during follow-up

### Comparison of the directions of effect in the final models

The range of the score depicting SWB (4–20 i.e., 16 different points) is four times the magnitude of range of HBSS (0–4). When comparing the magnitudes of effect, the negative HBSSchange had a greater effect (0.37 points) on SWB_2012_ than a negative SWBchange on HBSS_2012_ (4*0.072 points = *0.288 points)*. A positive HBSSchange resulted in slightly greater effect (0.31points) compared to a positive SWBchange (4*0.071 points = *0.284 points).* Thus, it appears that changes in HBSS have stronger effect on SWB than vice versa. The maximum effect of SWB_2003_ (range 4–20) on HBSS_2012_ (range 0–4) could be 16*0.02548 (i.e., -0.25 in Table 6) ≈ 0.41 and the maximum effect of HBSS_2003_ on SWB_2012_ was 4*0.3515 (i.e., -0.35 in Table 4) ≈ 1.4.

## Discussion

In the present study on 10,000 working-age Finns, a change in health behavior appears to have a slightly stronger effect on subsequent SWB than vice versa when adjusting for age, gender, education, diseases, baseline level of outcome, and negative life events prior to the follow-up. In addition, it was observed that on average both health behavior and SWB improve during the 9-year follow-up.

In both directions of effect, change in the predictor had a statistically significant effect on the outcome. When comparing the effect of change to the effect of baseline level, the baseline level of health behavior and SWB seem to be stronger predictors in the models than their changes. The effect of health behavior change (assessing only the direction of change) in the 9-year follow-up on subsequent SWB was of approximately equal magnitude to the effect of one point difference in the baseline health behavior and therefore about a fourth of the maximum possible effect of baseline health behavior. The effect of SWB change (assessing only the direction of change) in the 9-year follow-up on subsequent health behavior was about three times greater than the effect of one point better baseline SWB but about a sixth of the maximum difference in the baseline SWB. Furthermore, adjusting for health behavior change strengthened the effect of baseline health behavior and adjusting for SWB change strengthens the effect of baseline SWB.

Our results are in line with earlier research where increase in fruit and vegetable consumption has resulted in better life satisfaction even though the magnitude of the average effects was small. In 50,000 individuals in the UK, an increase of one portion of fruits and vegetables in a follow-up of 5-years resulted in a 0.133 point increase in well-being (*p* < 0.001, range of scale 0–36) [9]. In a follow-up of 2 years, an increased consumption of 8 portions of fruits and vegetables increased life satisfaction by 0.24 points (on a scale of 0–10), which, however, was estimated to be of similar magnitude to the effect of moving from unemployment to employment [10]. In earlier studies, bidirectional relationship of health or health behavior and SWB has been analyzed with the same method [22] and by cross-lagged panel models previously [23–25]. However, none of these studies explore the effect of intraindividual change.

The opposite directions of effects have distinct characteristics. When health behavior predicts SWB, an action-based characteristic is predicting a subjective evaluation and perception of life. Thus, it is reasonable that negative life events can have a significant effect on SWB, which was true in the present study. In contrast, negative life events did not affect subsequent health behavior. Age showed a significant effect on subsequent life satisfaction when adjusted for sociodemographic factors, health behavior and major diseases, which is in line with earlier research [1, 26]. However, age was not a significant covariate in our studies when life satisfaction predicted health behavior which also has been less explored in research. For example, the consumption of fruit and vegetables has shown a reversed U-shaped curve with the highest consumption around the age of 60 years [9]. In our study, the observed U-shaped pattern in health behavior by age could be caused by SWB that affects health behavior and also follows a U-shaped curve by age. This could imply that age in itself does not determine health behavior. Gender was a significant covariate when SWB predicted health behavior but not in the opposite direction. Personality, genetics or family upbringing, could affect both health behavior [27–30] and SWB [31]. However, these effects should stay constant during follow-up [as in 9].

Change in health behavior appears to have a significant effect on subsequent SWB and vice versa. In addition to interindividual differences, changes within an individual had an impact on the relationship between health behavior and SWB. An explanation for improvement in health behavior along with SWB might go through enhanced self-efficacy, which has been reported as a result of increased positive affect in a behavior change intervention [32]. Improved health behavior, on the other hand, can improve health and therefore also potentially SWB. Thus, change in both or in either health behavior or SWB is beneficial, and can be used to motivate personal health promotion. A change in health behavior can lead to both reduced costs of healthcare and – through improved SWB – also to better productivity, which is an interest in political decision making [31]. As in previous studies on this data [25] the results could be generalized to the working-age population in Finland and presumably in other Western countries.

### Strengths and weaknesses

The present study is to our knowledge the first to explore the effect of individual change in the bidirectional setting of multiple health behaviors and a measure of SWB. Thus, it provides more understanding in the complex relationship between health behavior and SWB. A consistent survey procedure and large population-based sample yield solid results. There is adequate representation of covariate groups and individuals showing also negative changes in health behavior and SWB, who usually participate less in health-related surveys. In addition, a follow-up of 9 years is a long enough time perspective in an adult’s life. Thus, a statistically significant effect of that size after such a follow-up is considerable*.*

The study has also limitations. Attrition might have caused some overrepresentation of positive changes as well as better baseline levels of HBSS and SWB. The magnitude of change was not quantified. Only the direction of change was used in the analysis. Therefore, firm conclusions of the effect of a specific change cannot be drawn. The use of only two follow-up points might be subject to bias of momentary external factors. However, the large data could eradicate most of the effects of individual factors in people’s lives. The study is observational in nature. Thus, definite conclusions about causality cannot be made. The measures are obtained by self-report which especially for health behavior could be subject to reporting-bias [33]. Even if self-reported diseases lack the severity assessments, disease count is the most common instrument to measure the level of multimorbidity being also linked with various health outcomes according to a recent systematic review [20].

### Further research

Changes can be explored in predictor, outcome or in both. Using the same data and different approaches allowing bidirectional analyses provides more knowledge on this area. Change could be quantified in more detail than in the present study. Secondly, different indicators for SWB should be studied when assessing the role of change in its association with health behavior. Such studies could contribute to acknowledge the potential of SWB in health behavior interventions. Thirdly, future research is needed to identify which characteristics or factors affect the change to identify those who could benefit from SWB aspect in health behavior interventions*.*

## Conclusion

A change in health behavior predicts subsequent SWB and vice versa in a follow-up of 9 years. The effect of health behavior change on SWBs is slightly stronger than vice versa.

## Supplementary Information

**Additional file 1.** List of diseases and life events.

## Data Availability

The dataset analyzed is not publicly available due to the study data containing variables of personal and sensitive nature and hence, due to the present legislation of Finland and the General Data Protection Regulation (GDPR) of the European Union, cannot be made openly accessible inside or outside Finland. In special cases data is available from the authors on reasonable request.

## Abbreviations

HBSS: Health behavior sum score
HBSSchange: Change in health behavior sum score during follow-up
SWB: Subjective well-being
SWBscore: Subjective well-being score
SWBchange: Change in subjective well-being subgroup during follow-up
