# Supplementary material for: Changed health behavior improves subjective well-being and vice versa in a follow-up of 9 years
Source: Health Qual Life Outcomes. 2022 Apr 21;20:66. doi: 10.1186/s12955-022-01972-4 (PMC9027415; doi:10.1186/s12955-022-01972-4)
Supplement: Supplementary file 1 — Additional file 1. List of diseases and life events. [file 12955_2022_1972_MOESM1_ESM.pdf]

Additional file 1: **List of Diseases and Life Events**

Diseases were reported by the participants on the following survey question in the Health and Social Support study (originally in Finnish or Swedish):

Has a doctor ever said, that you have or had had any of the following conditions (yes/no):
Long term bronchitis or bronchiectasis
Lung asthma
Allergic rhinitis e.g. hay fever
High blood pressure
Hypertension
High cholesterol
Diabetes
Myocardial infarction or coronary thrombosis
Angina pectoris, i.e. chest pain caused by coronary artery disease
Atrial fibrillation or atrial flutter
Stroke
Other cerebrovascular accident
Peptic ulcer
Celiac disease
Liver disease
Kidney disease
Rheumatoid arthritis
Arthrosis
Sciatica
Fibromyalgia
Cataract or glaucoma
Migraine
Epilepsy
Brain injury
Meningitis or encephalitis
Other cerebral disease of neurological disease
Depression
Panic disorder
Eating disorder
Other mental disorder
Malignant tumor
Other chronic or severe disease, which?

Negative life events (21 items).

Participants were asked if any of the following life events had happened (yes/no) during the previous 6 months/5 years/ earlier/ not at all. Participants also rated the happening as not so not so burdensome, burdensome, or extremely burdensome. [21,31]

Death of spouse
Death of own child
Death of mother
Death of farther
Dealth of a close relative
Death of a close friend
Severe illness of a familymember
Abortion (own or spouse's)
Misscarriage (own or spouse's)
Major difficulties with employer (Huomattavat vaikeudet esimiehen kanssa?)
Major difficulties with co-workers
Financial hardships (Oman taloudellisen tilanteen huomattava vaikeutuminen)
Divorce or separation
Increase in relational problems with spouse
Breakdown of a longterm friendship
Loss of one's job
Retirement
Unemployment of spouse
Illness couasin work disability of over 21 days
Severe injury
Being victim of physical, psychologiacal, or sexual violence.
